# *Syzygium cumini* (L.) Skeels improves metabolic and ovarian parameters in female obese rats with malfunctioning hypothalamus-pituitary-gonadal axis

**DOI:** 10.1186/s13048-019-0490-8

**Published:** 2019-02-04

**Authors:** R. O. A. Benevides, C. C. Vale, J. L. L. Fontelles, L. M. França, T. S. Teófilo, S. N. Silva, A. M. A. Paes, R. S. Gaspar

**Affiliations:** 1 Departamento de Ciências Fisiológicas, Universidade Federal do Maranhão, São Luís, Maranhão Brazil; 2 Departamento de Ciências Animais, Universidade Federal Rural do Semi-Árido, Mossoró, Rio Grande do Norte Brazil; 3 Institute of Cardiovascular and Metabolic Research, School of Biological Sciences, University of Reading, Harborne Building, Reading, UK

**Keywords:** Obesity, Ovary, Syzygium cumini, Polycystic ovary syndrome, Hyperinsulinemia, L-glutamate monosodium

## Abstract

**Background:**

Obesity is a chronic and multifactorial disease characterized by increased adipose tissue. In females, obesity leads to reduced ovulation and lower chances of conception in diseases like polycystic ovary syndrome, making it important to characterize complementary medicine to attenuate such deleterious effects. Therefore, the aim of this study was to assess the effects of a hydroethanolic extract from *Syzigium cumini* leaves in female reproductive impairments present in the obesity model of neonatal L-monosodium glutamate injection.

**Methods:**

Newborn Wistar rats received saline (CTRL) or L-monosodium glutamate 4 mg/g BW (MSG). At 90 days of age, CTRL and some MSG rats received saline, while others received hydroethanolic extract of *S. cumini* leaves (HESc 500 mg/kg/day, MSG-Syz group) for 30 consecutive days. Estrous cycle was determined by daily vaginal washes. On days 26 and 28 of treatment, oral glucose tolerance test and blood collection were performed for biochemical assessment. At the end, animals were euthanized during estrous phase; blood was collected to measure sex hormones and organs collected for weighing and histological evaluation.

**Results:**

MSG-Syz showed reduced Lee Index, retroperitoneal fat pads and restored gluco-insulin axis. Moreover, HESc treatment reduced serum cholesterol levels when compared to MSG. Treatment with HESc did not restore the oligociclicity observed in obese animals, though MSG-Syz reestablished ovarian follicle health back to CTRL levels, with proliferating primordial follicles – these effects were followed by a decrease on periovarian adipocyte area.

**Conclusions:**

This is the first report to show the reversibility of the reproductive dysfunctions seen in MSG female rats through ethnopharmacological treatment. Moreover, it expands the use of HESc as a prominent tool to treat metabolic and reproductive disorders. Finally, we provide novel evidence that, without a functioning hypothalamus-pituitary-gonads axis, metabolic improvement is ineffective for estrous cyclicity, but critical for ovarian follicle health.

## Background

Obesity is a chronic and multifactorial disease whose etiology stems mainly from the imbalance between daily energetic consumption and usage with basal metabolism, although genetic and environmental factors are also involved. According to recent estimates there were 1.9 billion overweight individuals over 17 years old in 2014, representing 39% of the world’s adult population while 42 million children under 5 years of age were overweight or obese in 2013 (for review see [1]). These data gain particular importance especially because obesity enhances cardiovascular risk factors, type II diabetes, neurological diseases, cancer and metabolic disorders [1], as well as fertility and reproductive disorders in females [2].

The ovulatory cycle is maintained by the functional and temporal integration of hypothalamus-pituitary-gonads (HPG) axis. The ovulatory function begins with pulsatile secretion of gonadotropin releasing hormone (GnRH) through the hypothalamus. GnRH stimulates the pituitary to release two important gonadotropic hormones: follicle stimulating hormone (FSH) and luteinizing hormone (LH), which act on granulosa and theca cells in the ovary by stimulating the synthesis of estrogens and androgens, respectively. In obese women, elevated serum levels of insulin and leptin cause disruption of this axis via ovarian and hypothalamic actions of these hormones [3]. Insulin directly stimulates androgen synthesis in the ovaries, acting in synergy with LH to increase the production of androgens in theca cells, being considered a co-gonadotrophin [4]. In turn, leptin stimulates neurons producing kisspeptin, a neuropeptide that enhances GnRH release [5].

High evolutionary conservation of reproductive function between mammals allows a parallel to be drawn between rodents and humans [6]. Rats with obesity induced by high fat diet presented early sexual maturity with greater follicular development, but with an accelerated rate of follicular loss and reduced fertile life. In addition, these animals presented more atretic follicles, suggesting that obesity may stimulate follicular apoptosis [7]. Similar to rodents, clinical and epidemiological investigations have shown that excessive fat accumulation leads to irregular menstrual cycles and infertility [8], while being associated with precocious puberty [9] and polycystic ovary syndrome (PCOS) [10].

Several drugs have been used to treat infertility disorders. Treatment of Wistar rats with clomiphene, an ovulation-inducing drug, improved the number of pups per litter [11]. Additionally, it has been demonstrated in vitro that resveratrol inhibits the production of androgen by ovarian theca cells, being clinically relevant for conditions associated with hyperandrogenism, such as PCOS [12], an endocrine disorder commonly associated with hyperinsulinemia and metabolic syndrome [10]. Likewise, insulin-sensitizing agents have been used to reduce hyperinsulinemia, providing positive impacts on reproductive parameters [13].

In this scenario, it is believed that medicinal plants are important sources of new chemicals with virtuous therapeutic properties and fewer side effects [14]. Syzygium cumini (L.) Skeels (Myrtaceae), is a tree popularly known as jambolan in Brazil, jamun in India, black plum in Europe, among others, whose anti-diabetic effects have been widely studied, especially due to its low toxicity [15]. Studies have indicated that ethyl acetic and methanolic extracts from *S. cumini* seeds have anti-diabetic properties in rats with streptozotocin-induced diabetes [16]. In addition to reducing glycemia, we showed that a polyphenol-rich extract prepared from *S. cumini* leaves restored peripheral glucose tolerance while inducing insulin secretion [17]. Of note, we have recently characterized the female L-monosodium glutamate (MSG) obese rat as a novel model to investigate the reproductive repercussions of obesity without interference of HPG axis [18].

Thus, taking into consideration the epidemiological importance of obesity and its repercussions on female reproductive system, in the present study we sought to expand the applicability of a hydroethanolic extract of *S. cumini* leaves (HESc) to female reproduction disorder induced by the MSG obesity model. Additionally, by using MSG-obese rats, we were also able to assess the individual impact of obesity on reproductive function without a functional HPG axis, shedding new evidence on the inter-regulation of obesity on ovarian function.

## Methods

### Botanical material and hydroethanolic extract preparation

Leaves of *S. cumini* were collected on the beautiful campus of the Federal University of Maranhão (UFMA) in the city of São Luís, MA - Brazil. A sample of the plant was sent to the Herbarium of Maranhão (MAR) of the Department of Biology of the same University, catalogued under number 4.574. HESc was prepared exactly as described previously [17].

### Animals and obesity induction

Female adult *Rattus norvegicus*, Wistar line, were provided by UFMA animal facility. These females were mated with healthy males and their female pups were then submitted to the process of neonatal obesity induction with MSG as described previously [18]. Briefly, the pups received subcutaneous injections of either MSG (4 g/kg/day) or saline at same volume for 5 consecutive days. All animals were kept in polyethylene cages, lined with Xilana®, with food and water ad libitum, under a light/dark cycle of 12 h, at a temperature of 20 ± 2 °C. Experimental procedure was approved by UFMA Ethics Committee for Animal Use (CEUA) under number 016/13.

### Experimental design

Upon birth, neonatal female rats were divided in two groups: a lean group, which received saline injections (Lean, *n* = 9) and an obese group, which received MSG. At 90 days, adult MSG rats were randomized into two subgroups: MSG obese animals receiving saline (NaCl 0.9% 0.1 mL/100 g/day; MSG group, *n* = 9), and MSG rats treated with HESc at a concentration previously shown to improve metabolic parameters [18] (500 mg/kg/day; MSG-Syz, *n* = 9). Lean animals also received saline solution – treatment started at 90 days of age, for 30 consecutive days by daily gavage. Concomitant to treatment, their estrous cycle was determined daily by vaginal washes between 8:00 and 10:00 am. Both animals and their chow were weighted thrice a week. Lee index (∛ (body weight (g) / nasoanal length (cm)) ∙ 1000) is considered an adiposity index in animals [19] and was verified at the beginning and end of treatment. On the 26th and 28th days of treatment, the animals were fasted for oral glucose tolerance test (OGTT) and blood collection for glycemia, triglyceridemia and total cholesterolemia, respectively. After 30 days of treatments all animals were euthanized in estrous phase using ketamine (70 mg/kg) and xylazine (10 mg/kg) i.p. injection followed by exsanguination while in estrous phase within a maximum period of 7 days. Blood was collected for estradiol, testosterone and LH determination; liver, pancreas, retroperitoneal and visceral fat (which corresponds to periovarian fat), uterus and ovaries were collected and weighed. Ovaries and visceral fat were stored in 4% paraformaldehyde for histological analysis while liver was kept frozen for mensuration of liver fat protocols.

### Estrous cycle assessment

Estrous cycle was performed daily, always in the morning between 08:00 and 10:00 am by analysis of vaginal washes as previously described [20]. In this procedure, the predominant cell type in vaginal smears, whether nucleated epithelial cells, cornified cells or leukocytes, was determined. The proportion between these cells was used to determine the phases of estrous cycle in: proestrus, estrus, metaestrus and diestrus. Females were identified as having regular or irregular cycles, as well as prolonged estrus according to previously described criteria [18, 20]. Cycle duration was determined by counting the number of days between one estrus to another [21].

### Oral glucose tolerance test

On the 26th day of treatment, all animals were submitted to OGTT. Eight-hour fasting animals were submitted to a small tail cut to verify basal glucose level using a digital glucometer (Accu-Chek Active®; Roche Diagnostic System, Branchburg, NJ, USA). Then, animals were given oral glucose (4 g/kg body weight) by gavage. New blood aliquots were collected at times 15, 30, 60, 120 min after glucose administration.

### Serum biochemical and hormonal analysis

At the end of treatment all animals were anesthetized and blood collected through aortic puncture and serum stored at − 20 °C until analysis. Serum triglycerides and total cholesterol were determined by spectrophotometric test kits (Labtest®, Lagoa Nova, MG, Brazil). Blood glucose determination was performed using a digital glucometer and its respective reagent tapes (Accu-Chek Active®; Roche Diagnostic System, Branchburg, NJ, USA). Sexual hormones were extracted from the serum prior to the assay as described previously [18]. Briefly, serum and diethyl ether at 1:5 *v*/v was mixed. This mixture was centrifuged at 1000 rpm for 3 min to separate the surfaces. The upper layer (ether) was collected and the extraction process repeated in the remaining serum. After two extractions, the tube was brought to a water bath for total ether evaporation. Finally, PBS-0.1% Tween buffer was used to dilute the pellet of extracted sex hormones. Before the analysis, a precision curve was obtained with a coefficient value of r^2^ > 0.96. All samples were measured on the same run. To determine plasma levels of estradiol and testosterone, commercial kits were used in serum (Roche Diagnostics GmbH, Manheim, Germany).

### Liver fat measurement

Both extraction and measurement of liver fat were performed exactly as described before [17].

### Ovarian histology

Ovaries were removed, cleaned and fixed in 4% paraformaldehyde for 24 h and stored in 70% ethanol until processing. The right ovary was embedded in paraffin and cut into sections 6 μm thick and stained with hematoxylin-eosin (HE). Only follicles containing an oocyte were considered. The oocyte is around 20–30 μm in diameter; therefore, we analyzed one section at every 6 cuts to ensure a minimum distance of 36 μm, preventing multiple counts of the same ovarian follicle. The follicles were classified as: primordial follicles consisting of a flattened and non-uniform layer of granulosa cells around an oocyte; primary follicles had less than two layers of cuboidal granulosa cells; secondary follicles had an oocyte surrounded by at least two layers of cuboidal granulosa cells, with no visible antrum; antral follicles had an oocyte surrounded by several layers of cuboid granulosa cells and containing one or more antral spaces, cumulus oophorus and theca cell layer [22]. Healthy follicles had no deformation on their granulosa or theca cell layers or pyknotic nuclei within their cells. Atretic follicles were characterized by shrinkage or collapse, presenting granulosa cells with at least two pyknotic nuclei or, if antral, with granulosa cells invading the antrum. One researcher single-blinded to the group performed these histological analyses. For statistical analysis, we divided the total number of each follicle type per ovary by the number of sections analyzed within that same ovary, therefore obtaining a mean number per section in order to avoid any bias regarding ovary size.

### Adipocyte histology

Periovarian fat deposits were processed together with the right ovary and stained with HE. At least 55 adipocytes were analyzed from each animal in 2–3 fields. All sections were selected at least 100 μm apart if the required number of fields were not sufficient in a single section. This distance prevented multiple analysis of the same adipocyte [23]. Photomicrographs were taken at a magnification of 200X. The area of adipocytes was calculated using AxionVision (AxioVs40x64 V 4.9.1.0, Carl Zeiss GmBH Microscopy). Subsequently, we calculated the frequency of adipocytes of similar size within 500 μm^2^. One researcher single-blinded to the group performed these histological analyses.

### Statistical analysis

Results are expressed as mean ± S.E.M and the groups compared to each other by ANOVA, using Tukey as post-test. The differences were significant when *p* < 0.05. The analyses were performed using the statistical program GraphPad Prism version 7.03.

## Results

### Obesity onset and liver fat accumulation

**Fig. 1:**
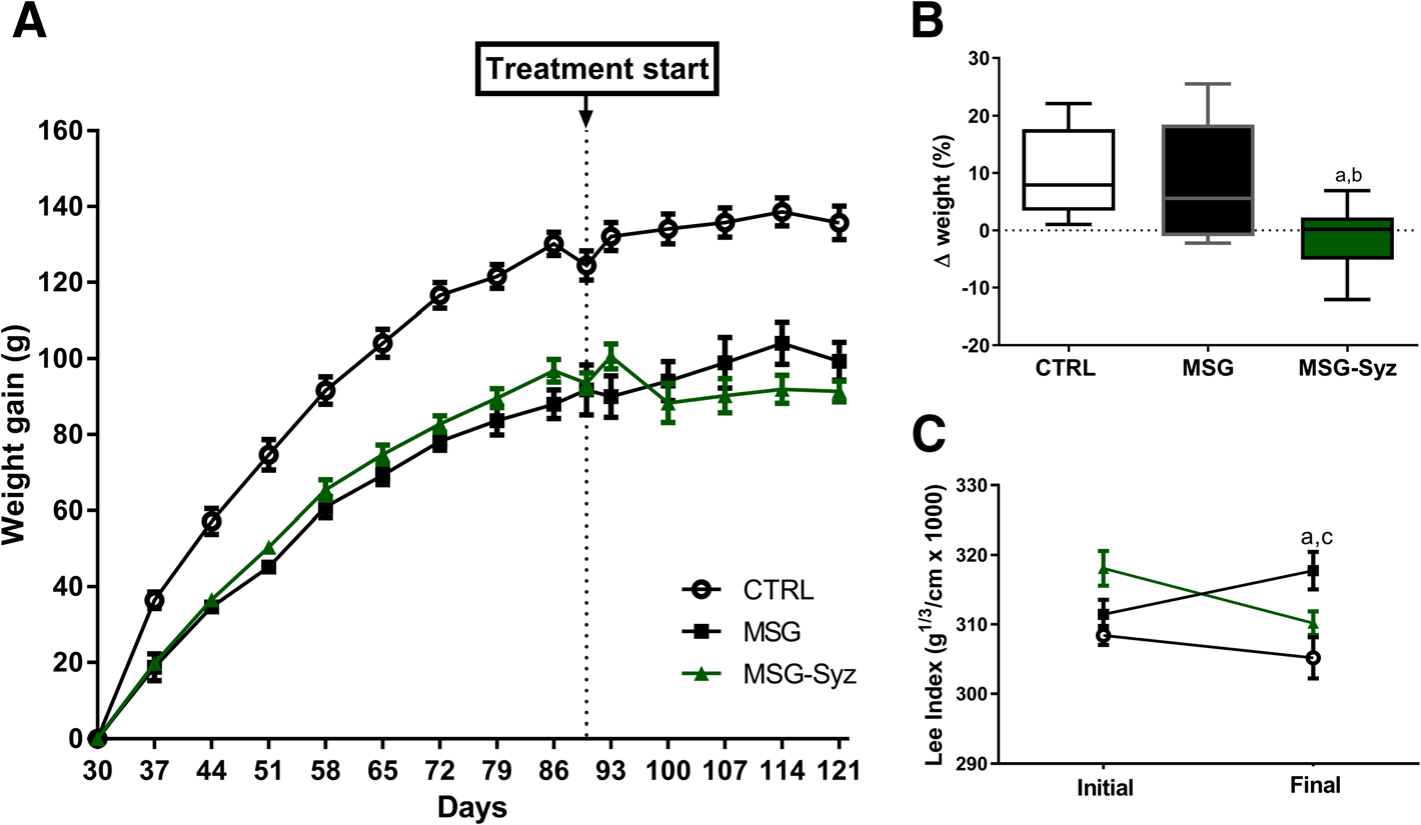
Weight gain and Lee Index are decreased by subchronic HESc treatment. Wistar lean (CTRL) and obese (MSG) rats received daily (v) isotonic saline administration, whereas some obese were treated with 500 mg/kg HESc (MSG-Syz), for 30 days. **a**: Weight gain curve of all groups measured weekly from weaning. **b**: Percentual delta change of body weight during treatment. **c**: Lee Index before and after treatment. In A and C values are expressed as mean ± SEM. In B data expressed as median, quartiles and range. *n* = 7–9 (*p* < 0.05). **a**: vs CTRL; **b**: vs MSG; **c**: vs MSG-Syz

HESc administration (500 mg/kg/day) promoted a marked weight reduction during the first 10 days of treatment, followed by stabilization (Fig. 1a). This was confirmed by the comparison of proportional weight gain between CTRL and MSG groups, which showed a trend to increase (9.64 ± 2.72 g and 7.91 ± 3.46 g, respectively), whereas MSG-Syz maintained their weight (− 1.10 ± 2.05 g, *p* < 0.05; Fig. 1b). Nonetheless, when analyzing the Lee Index, it became evident that HESc treatment restored body mass back to CTRL levels, whereas MSG animals continued to increase (Fig. 1c). As shown in Table 1, MSG-obese animals had significant increase in retroperitoneal and periovarian adipose tissue deposits compared to CTRL. Treatment with HESc reduced retroperitoneal fat deposition by 34.2%, an effect not observed on the periovarian adipose deposit (Table 1). Both reductions in Lee Index and fat deposition were not followed by changes on liver fat levels, probably because obesity induction was not sufficient to increase liver fat accumulation.

**Table 1:** HESc reduces fat accumulation without interfering with hepatic lipid profile of MSG-obese rats

	CTRL	MSG	MSG-Syz
Morphometric features			
Weight (g)	218.2 ± 6.67	181.7 ± 11.10^a^	161.1 ± 3.66^a^
Naso-anal length (cm)	19.71 ± 0.088	17.91 ± 0.269^a^	17.54 ± 0.102^a^
Retroperitoneal fat pads (g/100 g)	0.998 ± 0.078	3.470 ± 0.497^a^	2.283 ± 0.279^a. b^
Periovarian fat pads (g/100 g)	1.887 ± 0.126	4.008 ± 0.547^a^	3.964 ± 0.491^a^
Ovary (g/100 g)	0.073 ± 0.0049	0.052 ± 0.0055^a^	0.059 ± 0.0024^a^
Uterus (g/100 g)	0.292 ± 0.016	0.219 ± 0.024^a^	0.192 ± 0.014^a^
Liver (g/100 g)	3.41 ± 0.082	2.91 ± 0.131^a^	2.99 ± 0.210^a^
Hepatic lipid profile			
Total fat (mg/g Liver)	290.9 ± 38.27	298.4 ± 27.92	266.4 ± 42.49
Total Cholesterol (mg/g Liver)	2.20 ± 0.12	2.21 ± 0.07	2.16 ± 0.22
Triglycerides (mg/g Liver)	4.82 ± 0.21	8.32 ± 2.09	6.07 ± 0.73

Results expressed as mean ± S.E.M. *n* = 7–9. (*p* < 0.05)

^a^: vs CTRL; ^b^: vs MSG

### Glucolipid profile and glucose tolerance

**Fig. 2:**
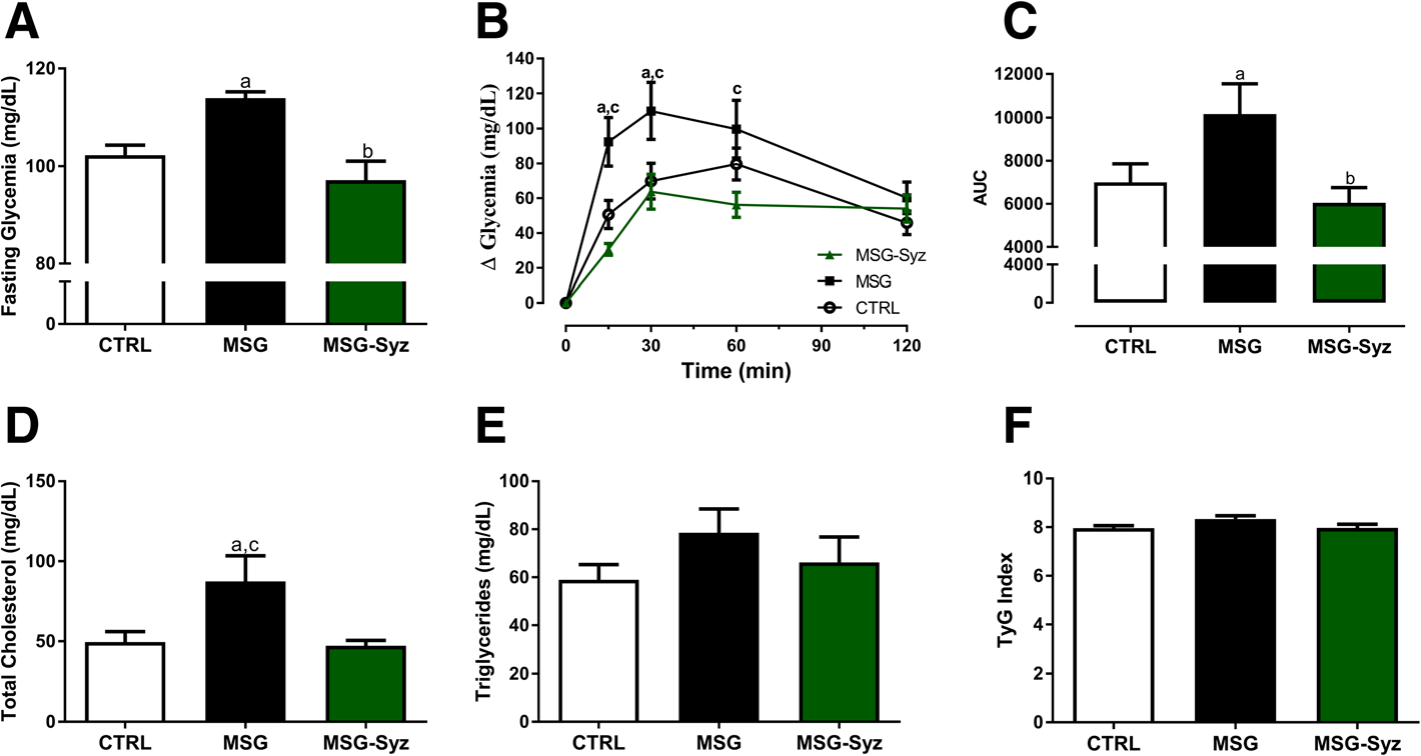
HESc reverses glucose intolerance cholesterol levels in MSG-obese female rats. **a**: Fasting glycemia measured after 8 h fasting. **b** After 8 h fasting, all groups received glucose (v.o) 4 g/kg. Glucose was measured at 0, 15, 30, 60 and 120 min by a thin tail cut. **c**: Area under the curve of OGTT. **d** and **e** Total cholesterol and triglycerides measured over fasting. **f** TyG index as a surrogate of insulin resistance. Formula can be checked on Material and Methods section. Values are expressed as mean ± S.E.M, *n* = 7–9 (*p* < 0.05). a: vs CTRL; b: vs MSG; c vs MSG-Syz

MSG rats presented higher fasting blood glucose, with glucose intolerance, whereas MSG-Syz showed a significant reduction on both parameters, reaching levels akin to CTRL (Fig. 2a-c). The same pattern was found on total cholesterol levels (Fig. 2d). Interestingly, no difference was found on both triglycerides levels and TyG Index (Fig. 2e-f), providing evidence that MSG-obese animals were not insulin resistant at the end of the study, despite having other comorbidities associated with metabolic syndrome.

### Estrous cycle and sexual hormones

**Fig. 3:**
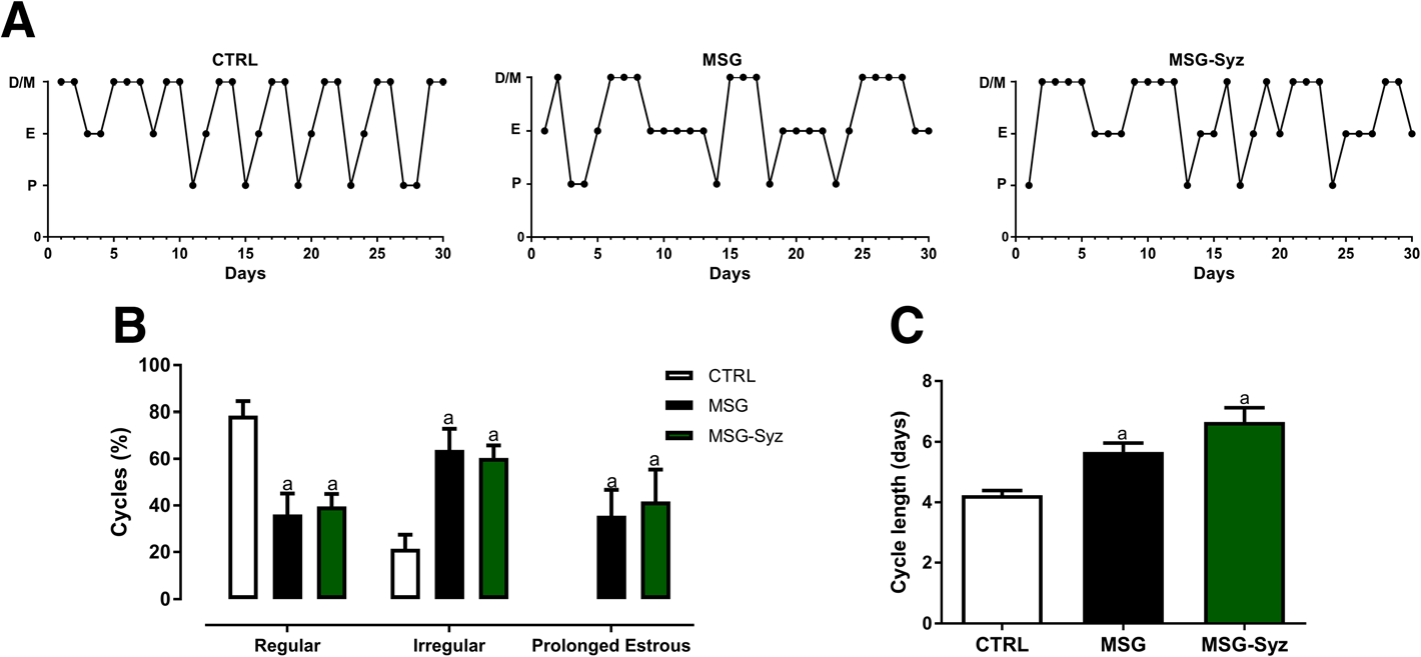
HESc does not interfere with estrous cyclicity or duration of MSG-obese rats. Vaginal washes were performed daily for 30 consecutive days. **a**: Representative estrous cycle of CTRL, MSG and MSG-Syz. **b**: Percentage of regular, irregular and prolonged estrous in all groups. **c** Cycle duration measured by number of days between consecutive estrous. Values are expressed as mean ± S.E.M., *n* = 7–9 (*p* < 0.05). a: vs CTRL

**Fig. 4:**
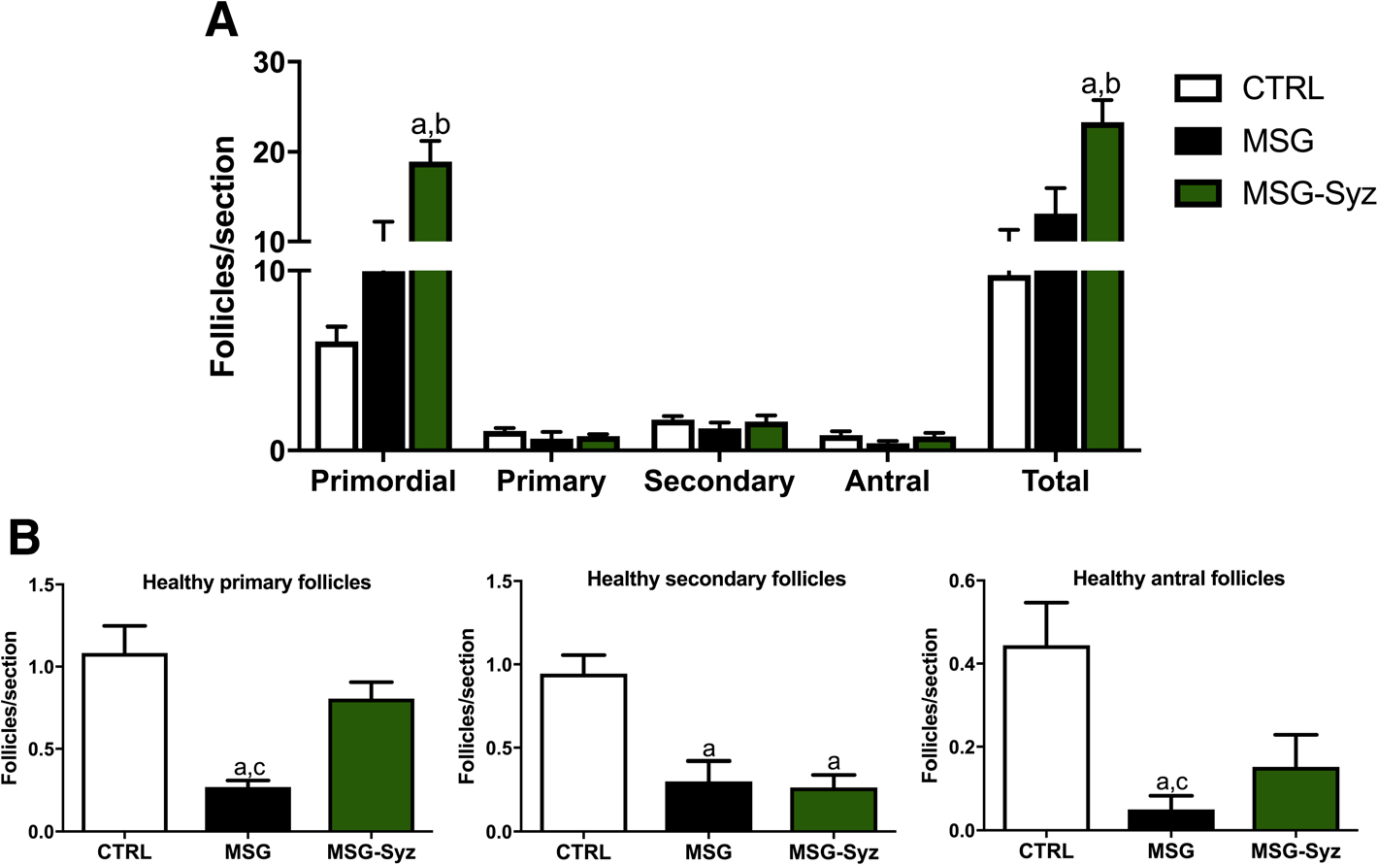
HESc promoted increased follicular production and restores follicle health in MSG-obese rats. At the end of treatment, the ovaries were removed, cleaned, fixed in 4% paraformaldehyde and submitted to histological material preparation procedure. The follicular rate per section was measured. **a**: follicles per section of each follicle type. **b**: number of healthy primary, secondary and antral follicles per section, respectively. Values are expressed as mean ± S.E.M., *n* = 7–9 (*p* < 0.05). a: vs CTRL; b: vs MSG; c: vs MSG-Syz

Considering the close association between obesity and reproductive dysfunction, estrous cycle was monitored throughout treatment. Figure 3 shows that, regardless of treatment, MSG animals were oligocyclic, unlike the normal cyclic profile observed in CTRL rats (Fig. 3a). The quantitative evaluation of cycles is shown in Fig. 4b. MSG and MSG-Syz groups presented a higher percentage of irregularity (63.89 ± 9.04%, 60.42 ± 5.4%, respectively) than CTRL (21.43 ± 5.99%) – only MSG animals had prolonged estrus. The induction of obesity resulted in longer cycles as well (MSG: 5.67 ± 0.3 days) in relation to control (CTRL: 4.24 ± 0.15 days). Despite the metabolic effects described above, treatment with HESc did not modify the pattern of cyclicity, nor did it reduce the duration of cycles in MSG-Syz (6.66 ± 0.45 days) (Fig. 3c). Hormonal dysregulation is often a cause of oligocyclicity, which led us to assess sex hormones important to the HPG axis. However, no difference was detected between group with regards to any of the hormones measured (Table 2).

**Table 2:** Subchronic HESc treatment does not alter sexual hormones of MSG-obese rats

	CTRL	MSG	MSG-Syz
Estradiol (pg/mL)	21.27 ± 3.66	23.93 ± 5.73	37.23 ± 8.43
Testosterone (ng/mL)	19.40 ± 1.94	18.54 ± 1.72	18.29 ± 1.40
Testosterone/Estradiol	1.06 ± 0.17	1.09 ± 0.47	0.62 ± 0.10
Dihydrotestosterone (pg/mL)	125.20 ± 15.42	88.52 ± 15.96	115.50 ± 18.75
Luteinizing Hormone (mIU/mL)	1.04 ± 0.15	0.68 ± 0.14	0.85 ± 0.13

Results expressed as mean ± S.E.M. *n* = 6–8

### Ovarian morphology

Despite the lack of effects of HESc on cyclicity and sexual hormone levels, it seems plausible that these might not reflect both quality and quantity of ovarian follicle maturation. Therefore, we evaluated ovarian follicular development and differentiation, as well as the amount and quality of each follicle type by light microscopy. As shown in Fig. 4a, MSG-Syz animals had a significant proliferation of primordial follicles, which then reflected on the total follicle number. Surprisingly, HESc treatment was able to improve follicle health both in primary and antral follicles (Fig. 4b). Such findings reinforce the follicular degeneration of MSG rats, while subscribing the beneficial effects that HESc promoted on ovarian morphology.

### Periovarian adipocyte histology

**Fig. 5:**
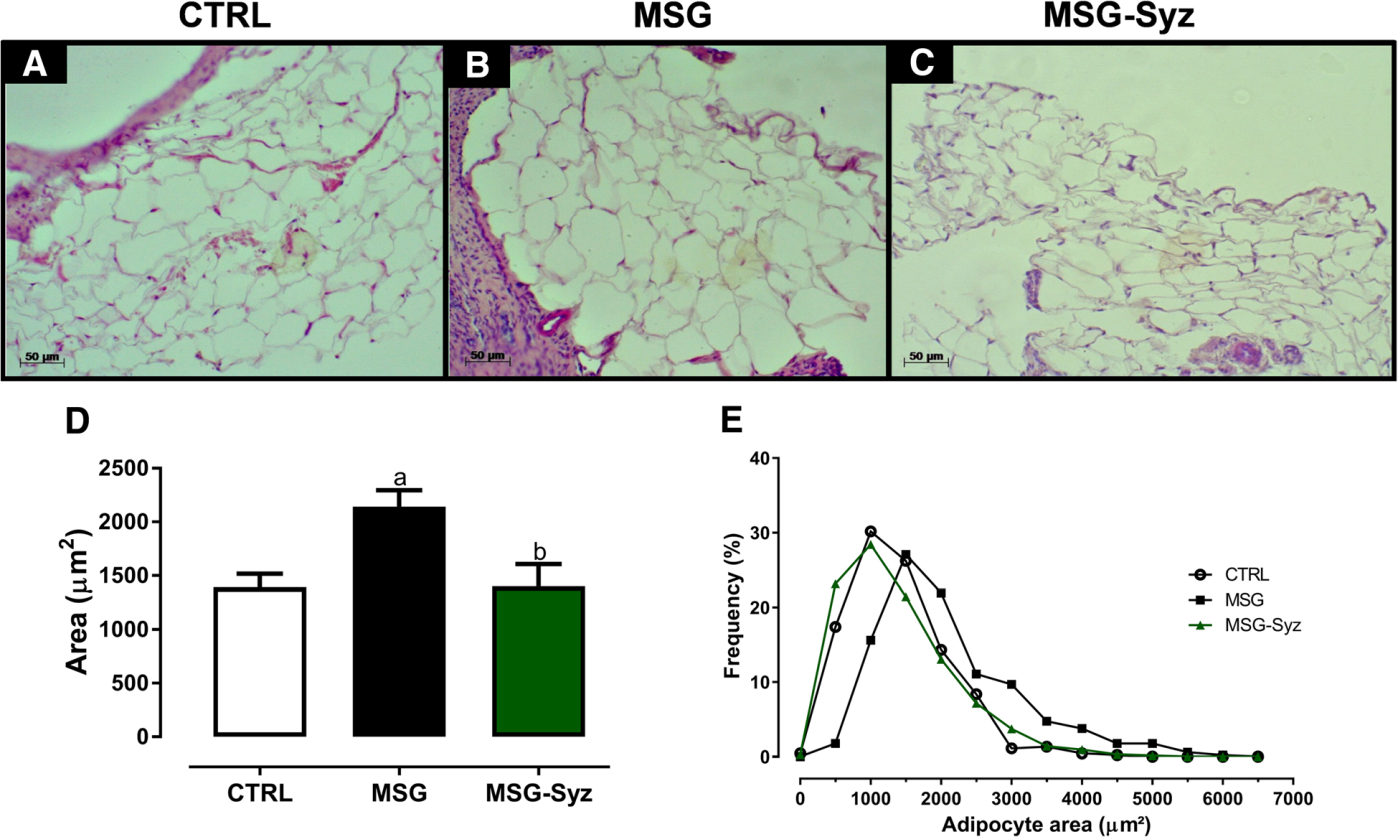
Periovarian adipocyte hypertrophy found in MSG rats is reversed by HESc treatment*.* Periovarian fat deposits were collected at the end of treatment and processed together with ovaries. These were then stained with HE and visualized under an optical microscope with a 200x magnification. Representative sections of CTRL (**a**), MSG (**b**) and MSG-Syz (**c**). **d**: Average area of periovarian adipocytes. **e**: Frequency of distribution of periovarian adipocyte area; the area of at least 55 adipocytes was measured in 2–3 different randomly selected fields. Values are expressed as mean ± S.E.M, *n* = 7–9. (*p* < 0.05) **a**: vs CTRL; **b**: vs MSG

To assess whether local adipocyte dysfunction was related to the ovarian recovery seen in MSG-Syz, we analyzed periovarian adipocytes. In addition to obesity and fat accumulation, MSG rats presented adipocyte hypertrophy in their periovarian adipose tissue as revealed by histological analysis when compared to CTRL (Fig. 5a-b). The quantitative histological analysis revealed that the MSG group had adipocytes with higher mean area (2143 ± 151.5 μm^2^) in contrast with CTRL (1391 ± 126.5 μm^2^; Fig. 5d). Interestingly, HESc restored the mean adipocyte area of MSG-Syz group to numbers comparable to CTRL (1402 ± 206.0 μm^2^; Fig. 2c-d), with complete reversal of the hypertrophy observed in MSG rats. In agreement, the frequency distribution of adipocyte area showed that the MSG group curve was shifted to the right, while the MSG-Syz group presented distribution similar to CTRL (Fig. 2e). These data indicate a role for periovarian adipose tissue in regulating ovarian function.

## Discussion

Female obesity leads to HPG axis disorders, resulting in reduced oocyte quality, endometrial receptivity and infertility [24]. Thus, therapeutic measures of adiposity reduction and improvement of the metabolic profile have shown a positive correlation with recovery of ovarian morphology and reproductive health [25]. At the same time, up until now there was no experimental model to address metabolic dysfunction without contribution of the HPG. In the present study, we have demonstrated that administration of HESc to obese MSG rats reduced total and periovarian adiposity, while restoring serum glucose and cholesterol levels with a relevant effect on glucose tolerance. These metabolic effects resulted in a significant recovery of ovarian follicle atresia, without improvement of estrous cycle, thus providing for the first time evidence that metabolic improvement have a positive impact on intraovarian environment regardless of HPG axis.

We have previously shown that neonatal MSG administration leads to a PCOS-like phenotype in young adult female rats [18]. Additionally, we and others have demonstrated the beneficial effects of HESc on metabolic parameters of obese and diabetic animals [17, 26]. Therefore, MSG-Syz animals displayed improved metabolic features, with reduced body weight, Lee index and fat accumulation. Such effects have been extensively studied and ascribed to the flavonoid content of HESc, specifically to myricetin and quercetin identified on this extract [17, 26, 27]. In a study with onion hydroethanolic extract rich in quercetin, Moon et al. (2013) attributed to this flavonoid the antiobesity effects observed in rats fed a high fat diet, since there was suppression of preadipocyte differentiation and inhibition of adipogenesis via modulation of β-oxidation of fatty acids, thermogenesis and lipid metabolism [28]. In this way, we can suggest that the antiobesity effect observed here is likely due to the flavonoid constituents present in HESc.

HESc displayed an anti-hyperglycaemic effect, reducing glycemia levels by 15% in MSG-Syz rats, bringing it back to CTRL levels. Moreover, HESc completely restored peripheral glucose tolerance. Such antidiabetic effects are in agreement with previous literature on the matter. For instance, Anandharajan et al. (2006) demonstrated that methanolic extract of *S. cumini* increased the expression of glucose transporter type 4 (GLUT-4) in a PI-3-kinase-dependent manner, promoting the activation of peroxisome proliferator-activated receptor gamma (PPAR-γ) pathway - an effect correlated with increased GLUT-4 transcription and consequent uptake of glucose [29]. This mechanism is of paramount importance to the obesity model under study, since obese MSG rats have a reduced number of GLUT-4 transporters in insulin-sensitive tissues [30]. Added to this, another report showed that the hydroethanolic extract of *S. cumini* leaves improved superoxide dismutase activity in different tissues of diet-induced obese mice, attributing such therapeutic effect to the antioxidant properties of the extract evaluated [26]. Nonetheless, we have shown that the same extract used on the present study induces insulin secretion both in INS-1E pancreatic β cells and ex vivo islets [17] without significant toxicity in vitro (data not published). Thus, we can corroborate previous literature and infer that the significant improvement of glucose metabolism found on MSG-Syz rats is likely due to the above-mentioned mechanisms.

In addition to the effects on glucose metabolism, administration of HESc to MSG rats resulted in a significant reduction in serum cholesterol levels. The hypolipidemic effect of *S. cumini* has been evidenced in works carried out with different extracts of the seeds and fruits of this species, which have attributed this action to the inhibition of 3-hydroxy-3-methylglutaryl-CoA (HMG-CoA) reductase enzyme, a key enzyme of biosynthesis cholesterol [31, 32]. Therefore, the marked reduction on cholesterol levels seen in MSG-obese female rats is in agreement with previous literature, providing evidence not only that HESc effects are consistent in both genders but also advocating the use of female animals for preclinical studies of hypolipemiant drugs.

Obesity is commonly associated with dysfunctional HPG axis, causing increase on testosterone and LH levels, mainly due to hyperinsulinemia [4, 33]. In fact, most PCOS models display hyperandrogenism, with increased LH levels [34]. On the other hand, female MSG rats do not follow such pattern, because the neonatal injection of this chemical severely damages the median eminence and arcuate nucleus of hypothalamus, leading to an obese animal with low levels of growth hormone (GH) as well as all of the above mentioned HPG hormones [18, 35, 36]. In fact, we described this model as a cheap and feasible tool to investigate the impacts of metabolic syndrome on female reproduction without the interference of HPG axis [18]. Corroborating our previous data, MSG animals displayed unaltered sex hormones, while MSG-Syz treatment was not sufficient to effectively affect these. This could also explain the lack of effect HESc had on estrous cycle, given that the HPG axis is the main regulator of female cycle. Surprisingly, though, HESc administration improved follicular count with recovery of follicular atresia shown in primary and antral follicles of MSG-Syz animals. While other flavonoid-rich extracts have shown positive effects on metabolic disorders, to the best of our knowledge this is the first description of HESc having a positive impact on ovarian function. Likewise, due to the experimental model used in other studies, most interventions result in recovery of both estrous cycle, HPG axis and ovarian follicle health [37, 38], making this the first report to show a detachment between these features, possibly due to the unique characteristics found on MSG rats. Whether the positive effect of HESc on intraovarian environment is an early outcome that would predict improved reproductive function or an isolated effect without further consequences is yet to be established, being a limitation of the present report.

Periovarian adipose tissue has received increasing attention over the past few years. In fact, Wang et al. [39] recently described the importance of ovary fat pad to reproduction in lean mice. Likewise, several reports relate the hypertrophy of periovarian adipocytes to ovarian dysfunction in different models [40, 41]. Therefore, given the beneficial effect of HESc on ovarian follicle development, we sought to determine whether periovarian fat pad is in some way related to the abovementioned effects. MSG rats once again corroborated previous data, showing hypertrophied periovarian adipocytes, whereas MSG-Syz had adipocytes’ comparable to CTRL. It seems reasonably to suggest that the paracrine effects exerted by periovarian adipocytes are important to the ovarian microenvironment and the positive effect seen on ovarian follicles from MSG-Syz could be related to the reduction on periovarian adipocyte area. Such hypothesis should be further investigated in future works.

## Conclusions

As a whole, this work shows for the first time that the hydroethanolic extract of *S. cumini* leaves produces beneficial effects on the metabolic parameters of female MSG-obese rats, without improving their oligocyclicity. Even so, HESc administration improved ovarian follicle health – an effect in some measure due to reduction of periovarian adipocytes. Not less important, this is the first report to show the PCOS-like features of MSG-obese rats can be at least partially reversed by pharmacological treatment, providing novel evidence that, without a functioning HPG axis, metabolic improvement is ineffective for estrous cyclicity, but critical for follicle health. Future perspectives include longer or earlier treatments, which may result in greater benefits on reproductive parameters of MSG-obese female rats.

## Abbreviations

FSH: Follicle stimulating hormone
GH: Growth hormone
GLUT-4: Glucose transporter type 4
GnRH: Gonadotropin releasing hormone
HE: Hematoxylin-eosin
HESc: Hydroethanolic extract of *S. cumini* leaves
HMG-CoA: 3-hydroxy-3-methylglutaryl-CoA
HPG: Hypothalamus-pituitary-gonads
LH: Luteinizing hormone
MSG: L-monosodium glutamate
OGTT: Oral glucose tolerance test
PCOS: Polycystic ovary syndrome
PPAR-γ: Peroxisome proliferator-activated receptor gamma
